# A Shared Genomic Alteration Supported the Diagnosis of Two Tumors in the Remnant Pancreas 7 Years after Resection of Distal Cholangiocarcinoma

**DOI:** 10.70352/scrj.cr.26-0308

**Published:** 2026-07-25

**Authors:** Hanako Tamura, Hiroki Ueda, Yuko Kinowaki, Shu Kato, Hiroshi Shintaku, Daisuke Asano, Yoshiya Ishikawa, Takaki Furuyama, Eriko Katsuta, Keiichi Akahoshi, Sadakatsu Ikeda, Daisuke Ban

**Affiliations:** 1 Department of Hepatobiliary and Pancreatic Surgery, Graduate School of Medicine, Institute of Science Tokyo, Tokyo, Japan; 2 Department of Comprehensive Pathology, Graduate School of Medicine, Institute of Science Tokyo, Tokyo, Japan; 3 Department of Human Pathology, Graduate School of Medicine, Institute of Science Tokyo, Tokyo, Japan; 4 Department of Precision Cancer Medicine, Center for Innovative Cancer Treatment, Institute of Science Tokyo, Tokyo, Japan

**Keywords:** distal cholangiocarcinoma, pancreatic metastasis, late recurrence, remnant pancreas, pancreatoduodenectomy, comprehensive genomic profiling

## Abstract

**INTRODUCTION:**

Metastatic tumors to the pancreas account for approximately 2% of all pancreatic malignancies, and pancreatic metastasis from distal cholangiocarcinoma is extremely rare. Differentiating pancreatic metastasis of cholangiocarcinoma from primary pancreatic cancer is often difficult, as both typically present as adenocarcinoma with similar immunohistochemical features.

**CASE PRESENTATION:**

A 65-year-old man underwent subtotal stomach-preserving pancreatoduodenectomy (SSPPD) for distal cholangiocarcinoma. He was diagnosed with pT3N0M0, Stage IIB according to the UICC TNM classification, 8th edition. Seven years after surgery, a routine blood test revealed an elevated serum carbohydrate antigen 19-9 level. Abdominal MRI and CT demonstrated 2 small masses, each approximately 10 mm in diameter, in the remnant pancreas without evidence of distant metastasis. Both lesions were diagnosed as adenocarcinoma by endoscopic ultrasound-guided fine-needle aspiration (EUS-FNA), suggesting either pancreatic metastases from distal cholangiocarcinoma or primary pancreatic cancer in the remnant pancreas. Completion total pancreatectomy with splenectomy was therefore performed. Histopathological examination showed that both lesions in the remnant pancreatic tail were morphologically similar to the initial cholangiocarcinoma. However, the possibility of primary pancreatic cancer could not be completely excluded. Comprehensive genomic profiling (CGP) of the initial cholangiocarcinoma and the remnant pancreatic tumor revealed a shared *TP53* H214R alteration, supporting the diagnosis of pancreatic metastases from distal cholangiocarcinoma. In addition, CGP identified *BRCA1* loss and an *FGFR3* alteration in the remnant pancreatic tumor as potentially actionable findings, although their therapeutic relevance in cholangiocarcinoma remains uncertain.

**CONCLUSIONS:**

We report a rare case of multiple pancreatic metastases occurring 7 years after resection of distal cholangiocarcinoma. Evaluation of genetic alterations provided supportive evidence to aid in distinguishing metastatic disease from primary pancreatic cancer in the remnant pancreas. In addition, these findings also suggested potentially relevant therapeutic targets.

## Abbreviations

CA19-9
: carbohydrate antigen 19-9
CGP
: comprehensive genomic profiling
CTCAE
: Common Terminology Criteria of Adverse Events
ERCP
: endoscopic retrograde cholangiopancreatography
EUS
: endoscopic US
EUS-FNA
: endoscopic US-guided fine-needle aspiration
FDG
: fluorodeoxyglucose
MRCP
: magnetic resonance cholangiopancreatography
PARP
: poly (adenosine diphosphate–ribose) polymerase
SSPPD
: subtotal stomach-preserving pancreatoduodenectomy
VAF
: variant allele frequency

## INTRODUCTION

Metastatic tumors to the pancreas account for approximately 2% of all pancreatic malignancies,^1)^ and pancreatic metastasis from distal cholangiocarcinoma is extremely rare, with only a limited number of cases reported in the literature. The diagnosis of remnant pancreatic tumors after distal cholangiocarcinoma resection is often challenging because both metastatic cholangiocarcinoma and primary pancreatic cancer typically present as adenocarcinoma with similar immunohistochemical features.

Here, we report a rare case of multiple tumors in the remnant pancreas detected 7 years after resection of distal cholangiocarcinoma, in which evaluation of genetic alterations provided supportive information for the differential diagnosis and suggested findings with potential therapeutic relevance, along with a brief review of the literature.

## CASE PRESENTATION

**Fig. 1:**
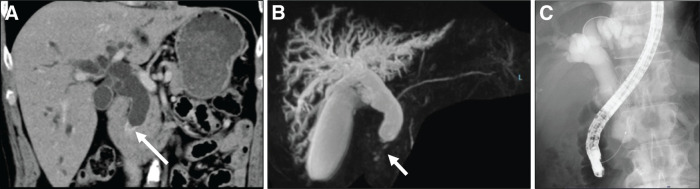
Preoperative imaging at the time of the initial surgery. (**A**) Contrast-enhanced abdominal CT demonstrating wall thickening of the distal bile duct. The arrow indicates the thickened distal bile duct. (**B**) MRCP showing dilatation of the common bile duct and intrahepatic bile ducts. The arrow indicates the dilated common bile duct. (**C**) ERCP revealing stenosis of the distal bile duct with upstream biliary dilatation. ERCP, endoscopic retrograde cholangiopancreatography; MRCP, magnetic resonance cholangiopancreatography

**Fig. 2:**
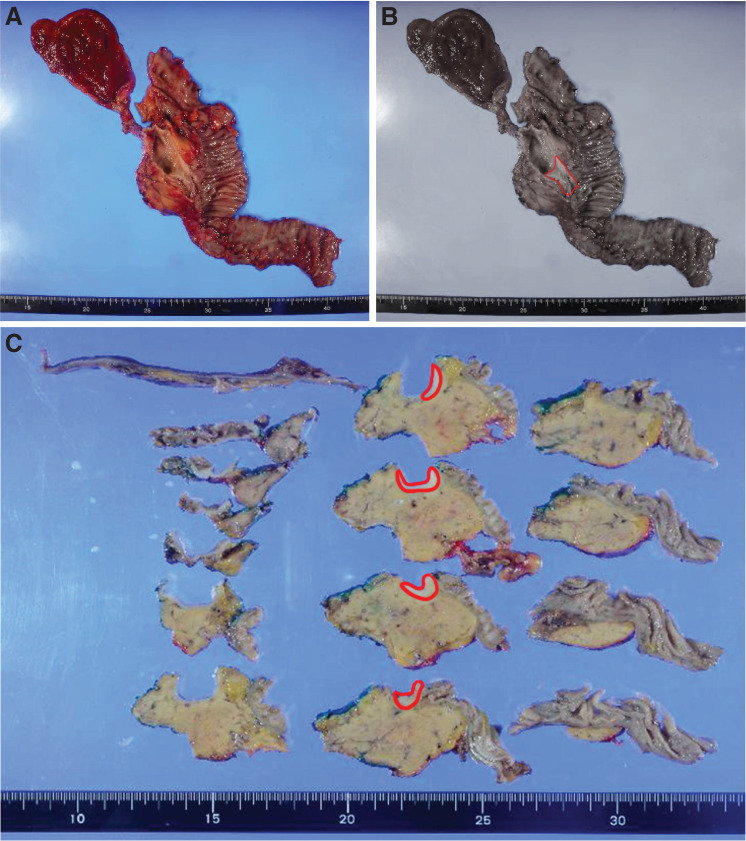
Resected specimen from the initial surgery. (**A**) Gross appearance of the specimen. (**B**) The red mark indicates tumor locations. (**C**) Cut surface showing a tumor extending from the distal bile duct to the ampulla of Vater. The red mark indicates tumor locations.

A 65-year-old man was diagnosed with distal cholangiocarcinoma. Preoperative imaging, including contrast-enhanced CT, MRCP, and ERCP, revealed a tumor in the distal bile duct with bile duct dilatation and no evidence of distant metastasis (**Fig. 1A**–**1C**). He underwent SSPPD. Intraoperative findings showed no distant metastasis or peritoneal dissemination, and an R0 resection was achieved. Pathological examination revealed adenocarcinoma (**Fig. 2A**–**2C**), and the pathological stage was determined to be pT3N0M0, Stage IIB, according to the UICC TNM classification, 8th edition. CGP using FoundationOne CDx (Foundation Medicine, Cambridge, MA, USA) had been performed at the patient’s request, which revealed a tumor harboring a *TP53* H214R mutation with an allele frequency of 5.5%. Adjuvant S-1 therapy was initiated after the initial surgery but was discontinued after 2 weeks because of CTCAE grade 3 enteritis. Subsequently, regular imaging and tumor marker follow-up were performed every 6 months, and no findings suggestive of any recurrence were observed during follow-up.

**Fig. 3:**
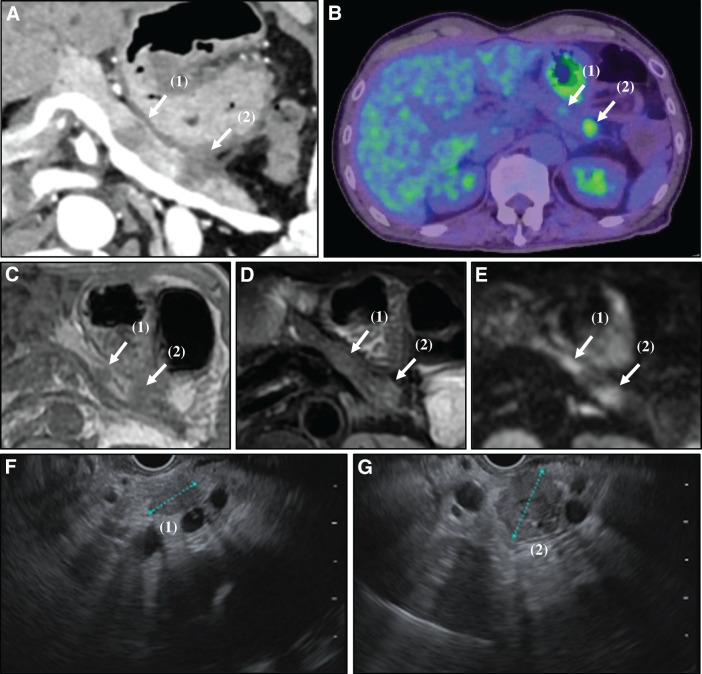
Imaging of remnant pancreatic lesions 7 years after initial cholangiocarcinoma resection. (**A**) Contrast-enhanced abdominal CT (arterial phase) demonstrating 2 hypovascular lesions in the remnant pancreatic tail. (**B**) FDG-PET-CT showing 2 areas of FDG uptake in the pancreatic tail tumors. MRI demonstrating 2 lesions in the remnant pancreas with low signal intensity on T1-weighted imaging (**C**), high signal intensity on T2-weighted imaging (**D**), and high signal intensity on diffusion-weighted imaging (**E**). (**F**, **G**) EUS demonstrating 2 hypoechoic masses in the remnant pancreas. White arrows indicate lesions (1) and (2), and blue dotted lines in panels (**F**) and (**G**) indicate their measured diameters. EUS, endoscopic US

**Fig. 4:**
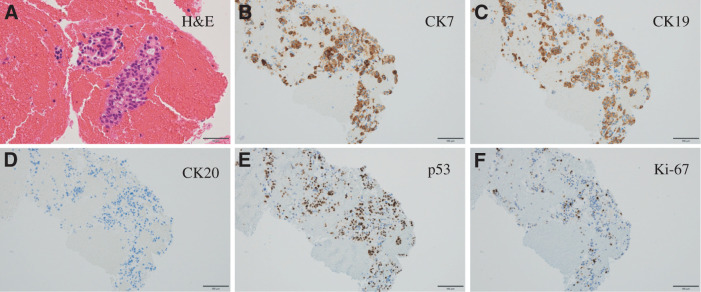
Histopathological findings of the pancreatic tumor obtained by EUS-FNA. (**A**) H&E staining showing features of adenocarcinoma. Immunohistochemical findings of CK7 (**B**), CK19 (**C**), CK20 (**D**), p53 (**E**), and Ki-67 (**F**). EUS-FNA, endoscopic US-guided fine-needle aspiration; H&E, hematoxylin and eosin

Seven years after the surgery, a routine blood test revealed an elevated serum CA19-9 level of 78.3 U/mL. Abdominal MRI and contrast-enhanced CT demonstrated 2 small masses, approximately 10 mm in diameter, in the remnant pancreas. FDG PET-CT showed increased uptake of FDG corresponding to these lesions in the remnant pancreas, without any evidence of other abnormal uptake (**Fig. 3A**–**3E**). EUS demonstrated 2 hypoechoic masses measuring 13 and 18 mm in the pancreatic tail (**Fig. 3F** and **3G**). Histological examination through EUS-FNA revealed adenocarcinoma in both lesions, which were morphologically similar to the initial cholangiocarcinoma (**Fig. 4A**). Immunohistochemical analysis showed positivity for CK7 and CK19 and negativity for CK20, with partial overexpression of p53 and a Ki-67 labeling index of approximately 10%–20% (**Fig. 4B**–**4F**). This profile supports a pancreatobiliary origin and argues against metastasis from other primary sites, as CK7 and CK19 are typically expressed in pancreatobiliary tumors, whereas CK20 is more commonly expressed in gastrointestinal tumors such as colorectal carcinoma. However, given the overlapping immunohistochemical profiles of pancreatic ductal adenocarcinoma and cholangiocarcinoma, differentiation between primary pancreatic cancer and pancreatic metastasis from distal cholangiocarcinoma remained difficult at this stage. Preoperative CGP using the EUS-FNA specimens was not performed because of concerns regarding the limited amount and quality of the specimens for comprehensive genomic analysis.

**Fig. 5:**
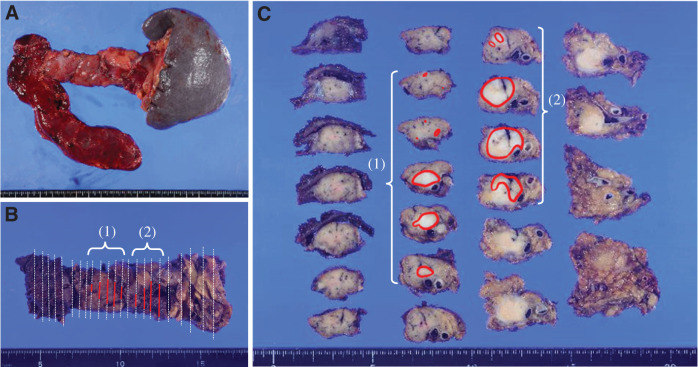
Resected specimen obtained from completion pancreatectomy. (**A**) Gross appearance of the resected specimen showing no obvious tumor exposure on the serosal surface. (**B**) Resected specimen with annotation indicating the site of sectioning. The red mark indicates tumor locations. (**C**) Cut surface of the specimen demonstrating 2 whitish nodules in the pancreatic tail. The red mark indicates tumor locations.

**Fig. 6:**
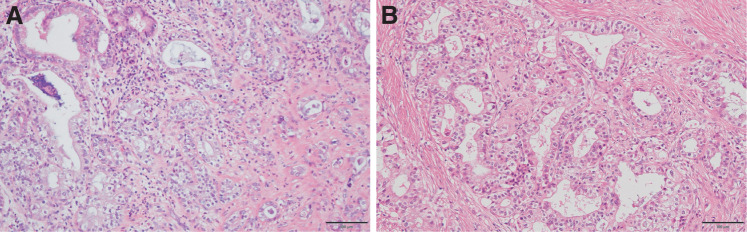
Comparison of histopathological findings between the initial cholangiocarcinoma and the remnant pancreatic tumors. (**A**) H&E staining of the initial cholangiocarcinoma showing a distal bile duct adenocarcinoma predominantly forming moderately to poorly differentiated tubular structures, with areas of fused glands and small nests in which the tubular architecture becomes indistinct. (**B**) H&E staining of the remnant pancreatic tumor showing a well-differentiated adenocarcinoma, exhibiting essentially the same histologic features. H&E, hematoxylin and eosin

The patient subsequently underwent completion total pancreatectomy with splenectomy. Macroscopic and histopathological examination of the resected specimen revealed 2 closely located but non-contiguous whitish nodules measuring 22 and 19 mm in the pancreatic tail, without serosal exposure (**Fig. 5A**–**5C**). Both lesions were histologically diagnosed as adenocarcinoma, presenting irregular glandular structures with a tendency for fusion and small nest-like growth patterns (**Fig. 6B**), resembling the initial cholangiocarcinoma (**Fig. 6A**). Perineural and venous invasion were observed in both lesions, and lymphatic invasion was identified in the tumor on the pancreaticojejunostomy side of the remnant pancreas, labeled as (1) in **Fig. 5B** and **5C**. Metastasis to a regional lymph node in No. 11 was also detected. Epithelial atypia in the pancreatic ducts was identified adjacent to the invasive component, whereas no neoplastic epithelial changes were observed in ducts distant from the tumor.

**Fig. 7:**
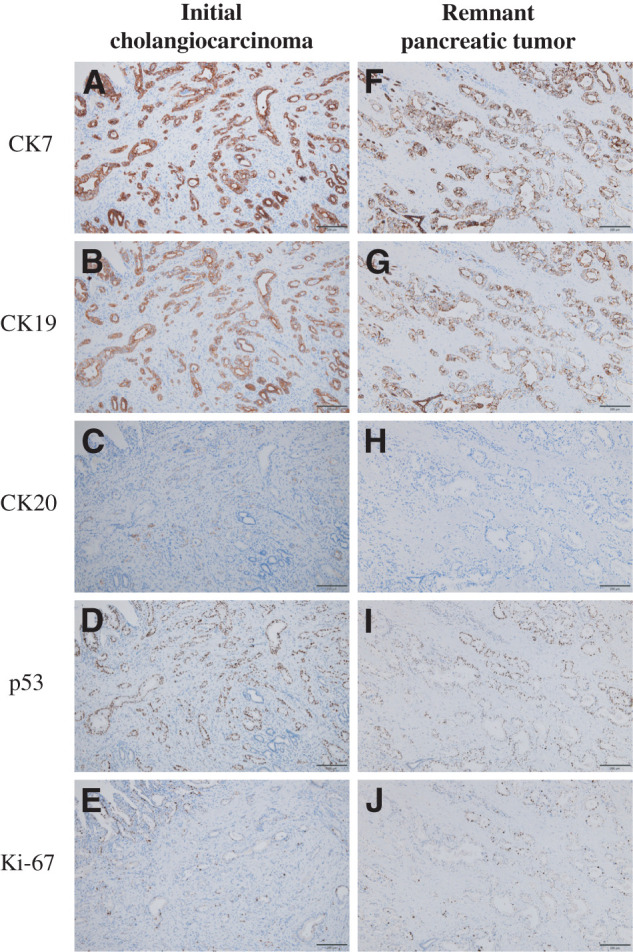
Comparison of immunohistochemical findings between the initial cholangiocarcinoma and the remnant pancreatic tumor. (**A**) Initial cholangiocarcinoma and (**F**) remnant pancreatic tumor of CK7 staining, those of CK19 (**B**, **G**), CK20 (**C**, **H**), p53 (**D**, **I**), and Ki-67 (**E**, **J**).

Immunohistochemical analysis showed positivity for CK7 and CK19 and negativity for CK20, with partial overexpression of p53 and a Ki-67 labeling index of 10%–20% (**Fig. 7F**–**7J**). Because the histological findings closely resembled those of the initial cholangiocarcinoma, additional immunohistochemical staining of the primary tumor was performed, which demonstrated similar staining patterns (**Fig. 7A**–**7E**). Differentiation between primary pancreatic cancer and pancreatic metastasis from cholangiocarcinoma remained difficult, as both tumors are known to exhibit similar immunohistochemical profiles.^2,3)^

Because a definitive diagnosis could not be established based on histopathological findings alone, we thought that genomic analysis might help in diagnosis. Therefore, CGP using FoundationOne CDx of the remnant pancreatic tumor was subsequently performed for diagnostic confirmation and therapeutic decision-making. The remnant pancreatic tumor showed a *TP53* H214R mutation, which was consistent with that of initial cholangiocarcinoma, at a higher allele frequency of 23%. In addition, *BRCA1* loss was detected, and *FGFR3* amplification was considered equivocal (**Table 1**). The presence of a shared *TP53* H214R mutation, which is not a common hotspot mutation site of *TP53*, supported a common origin. Based on these findings, we diagnosed the remnant pancreatic tumors as pancreatic metastases from distal cholangiocarcinoma.

**Table 1:** Comparison of gene panel results between the initial cholangiocarcinoma and the remnant pancreatic tumors

	Cholangiocarcinoma	Pancreatic tumor
Computational tumor purity	20%	32%
Biomarker findings		
Microsatellite status	Cannot be determined	Stable
Tumor mutational burden	Cannot be determined	8 muts/Mb
Others		HRD signature positive
Genomic findings	*TP53* H214R: 5.5%	*TP53* H214R: 23%
		*BRCA1* loss
		*FGFR3* amplification equivocal

The postoperative course was uneventful, and the patient was discharged on POD 14. Postoperative adjuvant chemotherapy was not administered after completion total pancreatectomy because of the patient’s previous intolerance to S-1 and his preference to avoid chemotherapy. He remains recurrence-free at 1 year after surgery.

## DISCUSSION

Differentiating pancreatic metastasis from cholangiocarcinoma and multiple primary pancreatic cancers arising in the remnant pancreas is clinically challenging. In the present case, the possibility of primary pancreatic cancer could not be excluded for the remnant pancreatic tumors; therefore, surgical resection was performed. If the lesions had been definitively diagnosed preoperatively as recurrence of distal cholangiocarcinoma, systemic chemotherapy would have been considered as a treatment option. However, the lesions were confined to the remnant pancreas without evidence of distant metastasis, and the disease-free interval after the initial surgery was 7 years. Therefore, surgical resection was considered an acceptable option for both diagnostic and therapeutic purposes.

Histopathological examination of the resected specimen initially raised the possibility of a primary pancreatic carcinoma, considering the long interval of 7 years after the initial surgery, the uncommon pattern of distal cholangiocarcinoma recurrence limited to the pancreas, and the presence of epithelial atypia in pancreatic ducts adjacent to the invasive component. However, neither neoplastic epithelial changes nor widespread precursor lesions such as PanIN were identified in pancreatic ducts distant from the tumors. In addition, the tumors showed morphological similarity and an overlapping immunohistochemical profile with the initial cholangiocarcinoma. These findings, in conjunction with genomic profiling results, supported the diagnosis of pancreatic metastasis from cholangiocarcinoma rather than multiple primary pancreatic cancers.

To date, a literature search of PubMed and the Ichushi-Web database identified only 3 reported cases of pancreatic metastasis after resection of biliary tract cancer^4–6)^ (**Table 2**). Among these, 2 cases were associated with pancreaticobiliary maljunction. In the report by Higashi et al.,^5)^ the recurrence was considered to be intraductal dissemination via reflux through the common channel. In the present case, no pancreaticobiliary maljunction was identified on preoperative imaging before pancreatoduodenectomy. Histopathological review of the specimen also revealed no findings suggestive of pancreaticobiliary maljunction. Possible mechanisms of recurrence, including intraductal dissemination and hematogenous spread, were considered; however, the exact route of metastasis could not be determined.

**Table 2:** Previously reported cases of pancreatic metastasis from biliary tract cancer

No.	Author	Year	Age (years)	Sex	Primary disease	Initial surgery	Pancreatobiliary maljunction	Site of recurrence	Pattern of recurrence	Time to recurrence	Diagnostic method
1	Matsubara et al.^4)^	2014	79	F	Ampullary carcinoma	Pancreatoduodenectomy	No	Pancreatojejunostomy and remnant pancreas	Local recurrence and intraductal dissemination	7 months	Molecular analysis
2	Higashi et al.^5)^	2017	49	F	Distal cholangiocarcinoma	Pancreatoduodenectomy	Yes	Remnant pancreas	Intraductal dissemination	1 year 6 months	Histopathological and immunohistochemical analysis
3	Kosuge et al.^6)^	2023	54	M	Gallbladder carcinoma	Extended cholecystectomy with extrahepatic bile duct resection	Yes	Remnant pancreas	Unclear	2 years 10 months	Histopathological and immunohistochemical analysis

F, female; M, male

With regard to the differential diagnosis between primary pancreatic cancer and pancreatic metastasis from biliary tract cancer, previous reports have relied mainly on histopathological and immunohistochemical findings. In the reports by Higashi et al.^5)^ and Kosuge et al.,^6)^ the diagnosis of pancreatic metastasis from biliary tract cancer was based on morphological similarity and concordant immunohistochemical staining patterns between the primary and metastatic lesions. Matsubara et al.^4)^ reported a case of ampullary carcinoma with recurrence at the pancreatojejunostomy and discontinuous intrapancreatic lesions 7 months after pancreatoduodenectomy. In that case, genetic mutation analysis and loss of heterozygosity analysis demonstrated a common origin between the recurrent and discontinuous lesions.

To the best of our knowledge, there have been no previous reports in which CGP provided supportive evidence for the diagnosis of pancreatic metastasis from cholangiocarcinoma. Therefore, the present case represents the first reported case in which CGP contributed to the diagnosis. *TP53* mutations are commonly observed in both cholangiocarcinoma and pancreatic tumors. However, in the present case, the specific *TP53* mutation of H214R was identical between the primary cholangiocarcinoma and the pancreatic tumors. According to the MSK-IMPACT study across a pan-cancer cohort,^7)^ *TP53* is the most commonly mutated gene (**Table 3**). Indeed, common hotspot mutations, such as R175, R248, and R273, occur at frequencies of approximately 1%–2% (**Table 4**); however, the frequency of the H214R mutation is as low as 0.14%. Because the H214R mutation is exceedingly rare, it is unlikely that the same mutation occurred in 2 different cancers. The VAF of this mutation was higher in the remnant pancreatic tumor than in the primary lesion (**Table 1**), although the VAF is influenced by tumor purity. Taken together, these findings provided supportive evidence that the remnant pancreatic tumors originated from the initial cholangiocarcinoma.

**Table 3:** Top 5 altered genes in the MSK-IMPACT cohort (n = 103367)

Frequency rank	Gene	Number of altered patients (%)
1	*TP53*	4437 (42.9%)
2	*KRAS*	1768 (17.1%)
3	*TERT*	1600 (15.5%)
4	*PIK3CA*	1354 (13.1%)
5	*APC*	1137 (11.0%)

**Table 4:** Frequency rank of *TP53* mutations in the MSK-IMPACT cohort (n = 103367)

Frequency rank	Mutation	Number of patients (%)
1	R175H	209 (2.02%)
2	R248Q	152 (1.47%)
3	R273H	137 (1.33%)
4	R273C	125 (1.20%)
5	R248W	113 (1.09%)
6	R213*	101 (0.98%)
7	R282W	97 (0.94%)
8	R342*	89 (0.86%)
9	G245S	75 (0.73%)
10	R196*	62 (0.60%)
11	Y220C	49 (0.47%)
12	X307 splice, X187 splice	48 (0.46%)
14	E285K	40 (0.39%)
15	R306*	36 (0.35%)
16	X126 splice	34 (0.33%)
17	V157F	31 (0.30%)
18	X332 splice, M237I	29 (0.28%)
20	G266R	28 (0.27%)
21	R273L	27 (0.26%)
22	P278S	26 (0.25%)
23	X261 splice	25 (0.24%)
24	X125 splice, Y163C, X225 splice, Y236C, H193R	24 (0.23%)
29	X331 splice, S241F, V173L, R158L, G245D	23 (0.22%)
34	H179Y, C176F, C238Y	22 (0.21%)
37	Q192*, R209Kfs*6, E286K	21 (0.20%)
40	E298*	20 (0.19%)
41	W91*, R249S, P151S	19 (0.18%)
44	R337C, G266E	18 (0.17%)
46	S127F, Y234C, R280K, R158H, H179R	17 (0.16%)
51	TP53 intragenic, V272L, V73Rfs*76, X224 splice, P152L, C135Y	16 (0.15%)
57	X33 splice, K132R, I195T, E294*, C277F	15 (0.15%)
62	H214R, X32 splice, R280T, Q331*, G154V, L194R, E271*	14 (0.14%)

Ranks are assigned based on mutation frequency. Variants with the same frequency are listed together and assigned the same rank.

The shared *TP53* H214R mutation supports the possibility that both tumors originated from a common ancestral clone. Because somatic mutations are generally maintained during clonal expansion, the presence of an identical mutation in both lesions supports a shared origin rather than independent tumor development. This is consistent with a model of clonal evolution, in which a subclone from the primary tumor may have persisted and later expanded in the remnant pancreas after a long latent period.

Although the interval of 7 years is relatively long, late recurrence has been reported in cholangiocarcinoma and may be associated with tumor dormancy, in which residual tumor cells remain quiescent for prolonged periods before reactivation.^8)^ In the present case, genomic alterations detected only in the metastatic tumors, namely *FGFR3* alteration and *BRCA1* loss, could either have been present at the time of the initial surgery but remain undetected because of limited tumor purity, or have been acquired later as a part of clonal evolution. However, we cannot help but speculate that these additional alterations are associated with the biological changes that favor escape from dormancy after a prolonged latent period.

Establishing the correct diagnosis is clinically important because therapeutic strategies between pancreatic cancer and distal cholangiocarcinoma differ significantly, although no recurrence has been observed in the current case to date. According to the National Comprehensive Cancer Network (NCCN) guidelines,^9,10)^ first-line treatment for recurrent distal cholangiocarcinoma consists of gemcitabine plus cisplatin in combination with immune checkpoint inhibitors, while oxaliplatin-based chemotherapy is recommended as a second-line regimen.^11–14)^ In contrast, treatment options for pancreatic cancer include gemcitabine-based or irinotecan-based combination regimens, depending on prior therapy.^15–17)^

Furthermore, in cases refractory to standard treatments, additional therapeutic options may be considered based on genomic alterations. In the present case, *BRCA1* loss suggests potential sensitivity to PARP inhibitors such as olaparib^18)^; however, the germline or somatic origin of this alteration could not be determined from tumor-only CGP, and therefore its therapeutic implications remain uncertain. Notably, *BRCA1* loss, as a representative alteration in homologous recombination repair genes, may be associated with increased sensitivity to platinum-based chemotherapy, which remains a key component of systemic therapy for biliary tract cancers, including regimens evaluated in recent trials such as TOPAZ-1 and KEYNOTE-966.^12,13)^ Furthermore, *FGFR3* alterations may provide eligibility for clinical trials of *FGFR* inhibitors,^19)^ as many currently available *FGFR* inhibitors target multiple *FGFR* family members, including *FGFR3*. Although most clinical evidence has been derived from urothelial carcinoma, *FGFR3* alterations have been associated with sensitivity to *FGFR* inhibition^20,21)^; however, evidence in cholangiocarcinoma remains limited.

## CONCLUSIONS

We report a rare case of multiple pancreatic metastases occurring 7 years after resection of distal cholangiocarcinoma. Although differentiation from primary pancreatic cancer in the remnant pancreas was challenging, evaluation of genetic alterations provided supportive evidence that contributed to the final diagnosis. These findings may also offer insights into potential therapeutic options.

## DECLARATIONS

### Availability of data and materials

The data supporting the findings of this study are available from the corresponding author upon reasonable request.
